# Choroidal and Retinal Thicknesses in Type 2 Diabetes Mellitus with Moderate Diabetic Retinopathy Measured by Swept Source OCT

**DOI:** 10.3390/biomedicines10092314

**Published:** 2022-09-18

**Authors:** Guisela Fernández-Espinosa, Elvira Orduna-Hospital, Ana Boned-Murillo, Maria Dolores Diaz-Barreda, Ana Sanchez-Cano, María Sopeña-Pinilla, Isabel Pinilla

**Affiliations:** 1 Aragon Institute for Health Research (IIS Aragon), 50009 Zaragoza, Spain; 2 Department of Applied Physics, University of Zaragoza, 50009 Zaragoza, Spain; 3 Department of Ophthalmology, Lozano Blesa University Hospital, 50009 Zaragoza, Spain; 4 Department of Ophthalmology, Miguel-Servet University Hospital, 50009 Zaragoza, Spain; 5 Department of Surgery, University of Zaragoza, 50009 Zaragoza, Spain

**Keywords:** diabetic retinopathy, type 2 diabetes mellitus, swept-source optical coherence tomography, choroidal thickness, retinal thickness

## Abstract

Background: To study choroidal thickness (CT) in type 2 diabetes mellitus (DM2) patients with moderate diabetic retinopathy (DR) and to correlate with changes in retinal thickness (RT) with swept-source OCT (SS-OCT) compared to healthy subjects. Methods: Fifty-four DM2 patients with moderate DR without diabetic macular edema (DME) and 73 age-matched healthy subjects were evaluated using SS-OCT to measure changes in total RT and CT in the nine areas of the Early Treatment Diabetic Retinopathy Study (ETDRS) macular grid. Results: The mean age was 64.06 ± 11.98 years and 60.79 ± 8.62 years in the diabetic and control groups, respectively. Total RT showed statistically significant differences in the temporal inner area, with higher values in the DM2 group (*p* = 0.010). CT did not show differences between the groups. There was a significant negative correlation between RT and age in all of the outer ETDRS areas and a positive significant correlation in the central area for the DM2 group. There was also a negative significant correlation between CT and age in all of the ETDRS areas except for the inferior inner area. In the DM2 group, a negative correlation was observed between RT and CT in the central area (*p* = 0.039) and in both horizontal parafoveal areas (temporal inner, *p* = 0.028; nasal inner, *p*= 0.003). Conclusion: DM2 patients with moderate DR have no changes with regard to CT. Both CT and RT decreased with age in DM2, showing a negative correlation between these factors in the central and horizontal parafoveal areas of the ETDRS grid.

## 1. Introduction

Diabetic retinopathy (DR), a diabetes mellitus (DM) microvascular complication, is one of the main causes of blindness worldwide. Its global prevalence is estimated to increase by 51% by 2045 [1].

Retinal vascular changes and increased permeability in the blood–retina barrier, as well as changes related to ischemia, are the main reasons for these findings in DR, including microaneurysms, edema, exudation, lack of perfusion, or retinal neovascularization leading to decreased visual acuity (VA). Not only the retina, but also the choroid, suffers from this vascular impairment with changes in its thickness and volume [2,3,4,5].

For proper function of the retina, a healthy choroid is required to nourish the different outer retinal layers, providing oxygen, nutrients, and thermoregulation [6]. Previously, the method used to study choroidal vascularization was indocyanine green angiography. This technique showed the blood flow and alterations in the choroidal vessels by injecting contrast dye into the bloodstream [7,8]. Recently, non-invasive techniques have been developed to assess both retinal and choroidal morphology and thickness, providing new insights into choroidal vascularization. Optical coherence tomography (OCT) is capable of acquiring high-resolution images of the different retinal and choroidal layers. Devices with longer wavelength lasers (1050 nm), such as swept-source (SS)-OCT, manage to overcome the high reflectivity of the RPE, increasing the visualization of choroidal vascularization, optimizing tissue penetration, and obtaining greater resolution and exploration speed [9,10].

Changes in retinal thickness (RT) in DM have been described as the thinning of the macular area before any sign of DR, mainly due to a decrease in the ganglion cell layer (GCL) and in the inner plexiform layer (IPL), both comprising the ganglion cell complex (GCC), and retinal nerve fiber layer (RNFL). Some studies show a significant decrease in RNFL thinning, suggesting neuronal damage related to diabetes, with neurodegeneration prior to the appearance of any retinal signs [11,12].

Choroidal changes are related to multiple ocular pathologies, such as age-related macular degeneration (AMD), pachychoroidal diseases, myopia magna, central serous choroidopathy, or inflammatory diseases, such as Harada’s disease [4,13,14,15]. CT increases or decreases depending on the ocular pathology, with modification in the choriocapillaris thickness.

Several authors have found that diabetic patients with DR experience a decrease in CT. Studies also show that as DR progresses, and with poor glycemic control, there is a greater decrease in CT. More research is needed to establish the degree of CT thinning in the different DM types and DR stages.

The aim of this study was to assess and correlate changes in both choroidal and retinal thicknesses in the macular area assessed by SS-OCT in type 2 DM (DM2) patients with moderate DR and without diabetic macular edema (DME) compared with healthy subjects.

## 2. Materials and Methods

### 2.1. Study Design

We performed a single-center cross-sectional study at the Ophthalmology Department of the Lozano Blesa University Hospital (Zaragoza, Spain) from February 2021 to December 2021, including a total of 127 eyes divided into two groups. Group 1 included 54 eyes of 54 DM2 patients with moderate DR according to the early treatment diabetic retinopathy study (ETDRS) classification (level 43 on the ETDRS retinopathy severity scale) [16] and without DME or other ophthalmological pathology that could compromise best-corrected VA (BCVA). Group 2 consisted of 73 eyes of 73 healthy subjects with no previous history of ocular pathologies or systemic diseases affecting the eye. The present study was approved by the local Ethics Committee for Clinical Research of Aragon (CEICA PI19/252), and the evaluation was conducted in accordance with the principles of the Helsinki Declaration. Detailed consent forms were obtained from each subject.

Exclusion criteria included amblyopia or BCVA less than 20/40 on the Snellen chart, refractive error over ± 5.50 diopters (D) of spherical equivalent (SE) or 3.00 D of astigmatism, intraocular pressure (IOP) higher than 20 mmHg, history of any ocular pathology affecting central vision, ocular hypertension, or glaucoma with perimetric involvement or papillary atrophy, or inability to perform good quality OCT (difficulty in layer segmentation, media opacification, or lack of fixation or cooperation).

### 2.2. Study Protocol

All participants underwent a complete ophthalmological evaluation, including BCVA expressed in logarithm of the minimum resolution angle (logMAR) measured with the 100% contrast to the ETDRS test, IOP measured by Goldmann tonometry, and axial length (AL) using an Aladdin KR-1 W Series optical biometry system (Topcon Corporation, Tokyo, Japan) as the mean of 5 measurements and expressed in mm. Clarus imaging was performed (Clarus 700^®^, Carl Zeiss Meditec AG, Jena, Germany) to examine and picture the eye fundus. In addition to the ophthalmological evaluation, a complete history was performed in which all aspects related to the patient’s disease (DM2) were collected, including current medication, time of diagnosis, glycosylated hemoglobin (HbA1c) levels, lipid levels, glomerular filtration, and creatinine levels. Values were obtained within less than 6 months of the examination.

**Figure 1:**
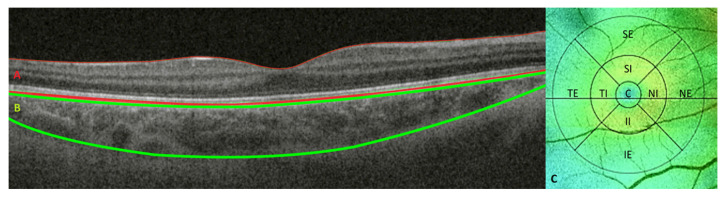
Total retinal thickness is between the red lines (**A**), choroidal thickness is between the green lines (**B**), and the nine areas of the ETDRS grid (**C**) measured by deep range imaging (DRI) Triton swept source (SS)-OCT for a right eye. (Abbreviations C, Central; SE, Superior External; TE, Temporal External; NE, Nasal External; IE, Inferior External; SI, Superior Internal; TI, Temporal Internal; NI, Nasal Internal; II, Inferior Internal).

SS-OCT was performed using deep range imaging (DRI)-Triton SS-OCT (Topcon Corporation, Japan) by the same explorer (G.F.-E.). RT and CT were automatically segmented with IMAGEnet 6 Version software 1.22.1.14101^®^ 2014 (Topcon Corporation, Tokyo, Japan); the segmentations were reviewed, and if any error was found, they were manually corrected, retested, or discarded. The image quality scale, which ranges from 0 to 100, should measure over 60. RT and CT values were measured in micrometers (μm) in the nine areas of the ETDRD grid with the 3D Macula protocol. The central (C) area was 1 mm in diameter; the outer or perifoveal ring was 6 mm in diameter divided into 4 areas: superior external (SE), temporal external (TE), nasal external (NE), and inferior external (IE); and the inner or parafoveal ring was 3 mm in diameter divided into 4 areas: superior internal (SI), temporal internal (TI), nasal internal (NI), and inferior internal (II) (Figure 1).

### 2.3. Statistical Analysis

Statistical analysis was performed using the Statistical Package for the Social Sciences software (SPSS version 20, SPSS Inc., IBM Corporation, Armonk, NY, USA). First, a descriptive and frequency-based analysis of the sample was carried out according to the demographic variables and clinical characteristics. Normal distribution of the values was studied with the Kolmogorov–Smirnov test, and subsequently, the Mann–Whitney U test was performed for independent nonparametric samples to assess if there were statistically significant differences between the groups. For the correlation of the anatomical results and the disease control parameters, a bivariate analysis was performed using Spearman’s rank correlation coefficient test. For all analyses, a value of *p* <0.05 was considered statistically significant.

## 3. Results

The mean age of the 54 DM2 patients was 64.06 ± 11.98 years (range 42–86 years) and 60.79 ± 8.62 years (range 42–83 years) for the 73 healthy controls. There were no age differences between groups (*p* = 0.082, Mann–Whitney U test). The gender distribution was 39.7% and 31.5% females and 60.3% and 68.5% males in the control and in the DM2 group, respectively, and there were no gender differences between groups (*p* = 0.339, Chi-square test). DM2 patients were well controlled, with a mean HbA1c of 7.58 ± 1.29%; the mean time since diagnosis was 2.50 ± 2.88 years (range 0–11 years). Glycemic, lipid, and renal function values are presented in Table 1.

There were no differences between groups in either AL (*p* = 0.075), SE (*p* = 0.110), or IOP (*p* = 0.676). BCVA taken with the 100% contrast of the ETDRS test reached statistical significance differences (*p* = 0.001), with lower VA in the DM2 group. Values are shown in Table 2.

### 3.1. Retinal and Choroidal Thickness Assessment

**Figure 2:**
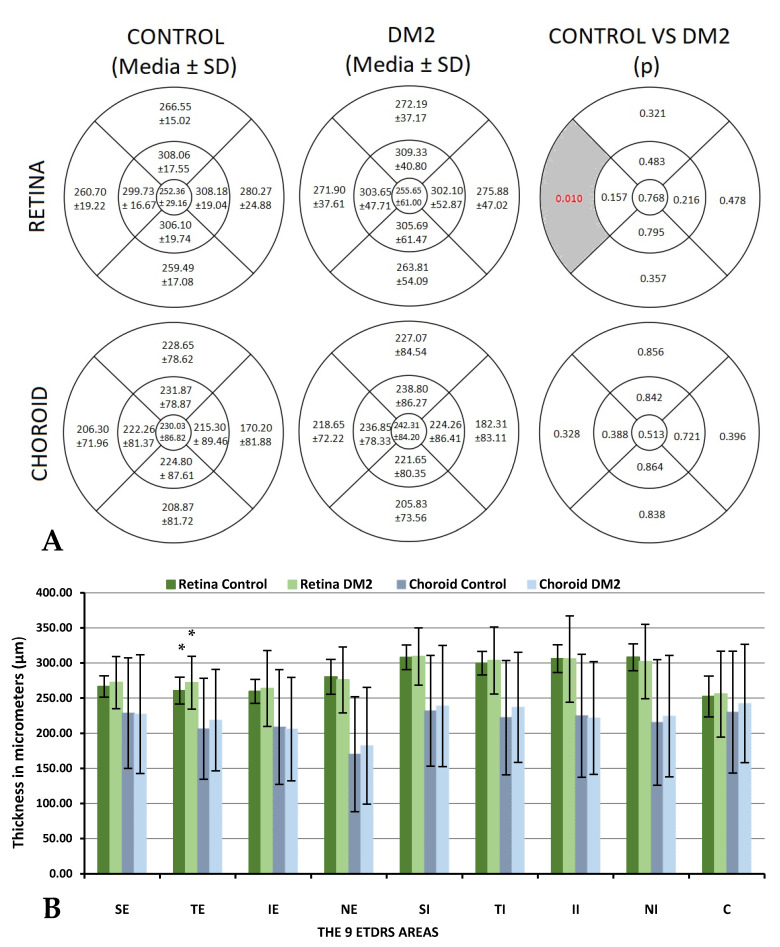
Mean and standard deviation (SD) of retinal and choroidal thicknesses measured using DRI-Triton SS-OCT in patients with type 2 diabetes mellitus (DM2) and in healthy controls and their comparison (*p*-value): (**A**) The measurements are represented in the nine areas of the early treatment diabetic retinopathy study (ETDRS) grid. The left quadrants represent the temporal quadrants, and the right quadrants represent the nasal quadrants. Differences that reached statistical significance (*p* <0.05) are shown in red with a grey background. (**B**) Retinal and choroidal thicknesses are represented in a bar chart. Statistically significant differences (*p* < 0.05) are marked with (*).

Looking for RT changes, statistically significant differences were found in the TE area, with thicker thickness in the DM2 group (260.70 ± 19.22 μm vs. 271.90 ± 37.61 μm with *p* = 0.010, in the control and DM2 groups, respectively). We observed greater variation in RT among the DM2 group, which had a higher SD. No statistically significant differences were found between the DM2 group and the control group in any of the nine ETDRS areas for CT. The values are presented in Figure 2.

### 3.2. Retinal and Choroidal Thickness Correlations

**Figure 3:**
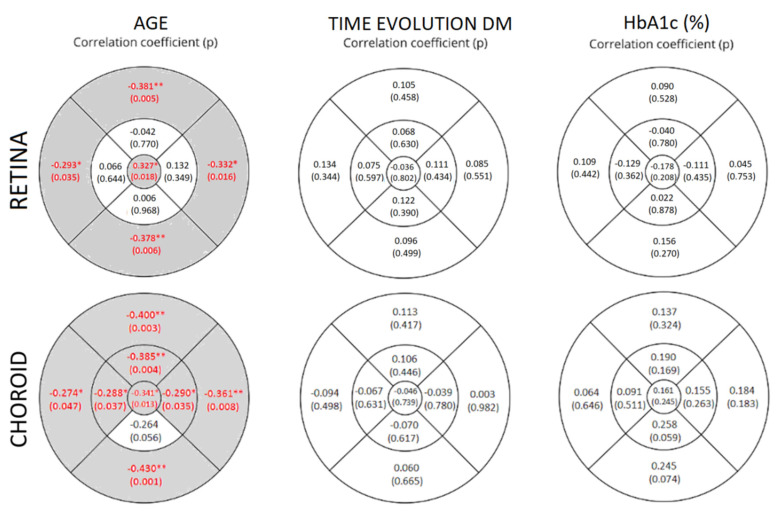
Correlation coefficients and statistical significance (*p*-value) of retinal and choroidal thicknesses with age, time of DM evolution, and glycosylated hemoglobin (HbA1c) levels (%) in DM2 patients. They are represented in the nine areas of the early treatment diabetic retinopathy study (ETDRS) grid. The left areas represent the temporal quadrants, and the right areas represent the nasal quadrants, as if a right eye was represented. The values that reached statistical significance (*p* < 0.05) are shown in red with a grey background and marked with (*) when *p* < 0.05 and with (**) when *p* < 0.01.

We used Spearman’s rank correlation coefficient test to correlate RT and CT with age, DM evolution time, and HbA1c levels (%) in all of the ETDRS areas (Figure 3). Studying the correlation between age and RT, a negative significant correlation (*p* < 0.05) was found in the four areas of the outer ETDRS ring, and a positive significant correlation (R = 0.327; *p* = 0.018) was found in the central ring. According to age and CT, there was a significant negative correlation (*p* < 0.05) in all of the ETDRS areas except in the II area (R = −0.264; *p* = 0.056). No significant correlations were found regarding the time of DM evolution and HbA1c for either RT or CT.

**Figure 4:**
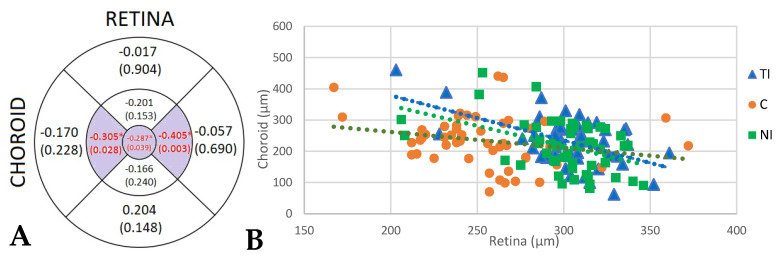
(**A**) Correlation coefficients and statistical significance (*p*-value) between choroidal thickness and retinal thickness in DM2 patients in the nine areas of the early treatment diabetic retinopathy study (ETDRS) grid. Left areas represent the temporal quadrants, and right areas represent the nasal quadrants, as if the figure represents a right eye. The values that reached statistical significance (*p* <0.05) are shown in red with a purple background and marked with (*). (**B**) Scatter plot with regression lines correlating retinal and choroidal thicknesses in temporal internal (TI), central (C), and nasal internal (NI) areas.

We also found negative significant correlations between RT and CT in TI (*p* = 0.028), C (*p* = 0.039), and NI (*p* = 0.003) areas in the DM2 group; in these areas, any increase in total RT was associated with a decreased CT (Figure 4).

## 4. Discussion

Retinal and choroidal layers undergo changes due to DM progression, especially in those patients with poor glycemic control and with longer evolution times. In addition to retinal and choroidal changes, there are many other ophthalmic manifestations associated with disease evolution, such as a diminution of BCVA as pathology progresses [17,18], an increased risk of cataract development [19], corneal changes such as corneal epithelial erosions, corneal endothelial cell damage, or endothelial decompensation with bullous keratopathy [20,21]. In addition, DM may be a risk factor for dry eye syndrome, with a disruption of the Meibomian gland function and other ocular surface manifestations [20,22]. Functional changes in diabetes related to neurodegeneration have also been found, such as alterations in color perception, reduction in contrast sensitivity (CS), and diminished dark adaptation, as DM progresses and is associated with diabetic neuropathy [23,24,25].

In our study, we analyzed a group of DM2 patients with moderate DR (level 43 on the ETDRS scale) without any sign of DME to evaluate changes in their RT and CT. The study was designed so that the groups were similar in terms of variables that could modify the results, in special CT, such as sex, age, or AL, and we included patients with IOP within the normal range, excluding glaucomatous patients.

Statistically significant differences were found in the 100% contrast BCVA measurement between the DM2 group and the control group (*p* < 0.001), with lower VA in the DM2 group (0.10 ± 0.12 LogMAR in the DM2 group vs. 0.04 ± 0.05 LogMAR in the control group); although, no sign of DME was observed. Authors such as Tiepei et al. [26] and Kim et al. [17] have found similar BCVA results (0.10 ± 0.19 LogMAR and 0.10 ± 0.13 LogMAR, respectively) studying DM2 patients, but they found no differences from their control groups. Wang et al. [18], in 2019, also found lower BCVA in DM2 patients than in the control group, which was even lower with the progression of DR. Lee et al. [27] studied three different groups, including a control group, DM1 group, and DM2 group. The authors found differences between all of them (*p* = 0.027), but these differences disappeared in post hoc analyses (control vs. DM1 group, *p* = 0.056; control vs. DM2 group, *p* = 0.088; DM1 group vs. DM2 group, *p* = 1). BCVA decreases with disease evolution time, probably due to the retinal neurodegeneration theory, which generates worse generalized visual function [28].

In our study, we did not find differences in RT between the DM2 group and the control group except in the TE sector (260.70 ± 19.22 μm in the control group vs. 271.90 ± 37.61 μm in DM2 patients, *p* = 0.010). We observed that there was greater variability in the RT measurements of the DM2 group, with a higher SD in the nine ETDRS areas than in the control group. Jiang et al. [29] also found differences in the TE sector between healthy subjects and DM2 without DR (326.0 ± 14.4 μm vs. 319.9 ± 16.7 μm with *p* = 0.01, in the control and DM2 groups, respectively); they found differences in other sectors, including the TI area (*p* = 0.02) and the C sector (*p* = 0.04), with RT being thicker in the control group. Other authors have found an increase in RT in DM patients, such as Orduna et al. [11], who found a thicker RT in the DM1 group (253.11 ± 28.36 μm vs. 239.77 ± 22.91 μm, DM1 vs. control; *p* = 0.014). In addition, Lattanzio et al. found similar results, with an increase in thickness measured by OCT in both DM1 and DM2 patients, being even thicker as DR progresses [30].

Studying CT, no statistically significant differences were found between groups in any of the nine ETDRS areas, but we can appreciate that CT is thicker in the DM2 group. Authors such as Orduna et al. [4] have also studied CT in DM1 patients with no DR and healthy subjects and found differences in some of the areas, including C (*p* = 0.025), SE (*p* = 0.040), IE (*p* = 0.036), SI (*p* = 0.014), and II (*p* = 0.016), with a thicker CT in the DM1 group. Wang et al. [31] found that CT increased in the early DR stage and further decreased with DR progression, especially in proliferative severe DR. Laíns et al. [32] also found a thicker CT in DM2 patients in the early stages of DR than in the control group, and they found that CT decreased in advanced stages of DR. Horváth et al. [33] also found a thinner choroid in patients with proliferative DR, suggesting that DM itself and DR progression significantly affect CT. In 2018, Abadia et al. [5] also found that DM2 patients presented a thinner CT than healthy participants.

We found a significant negative correlation between RT and the age of DM2 patients in the external ETDRS ring; older patients showed a decrease in their RT. On the other hand, we found a significant positive correlation between RT and the age of DM2 patients in the C ETDRS area, which increased with age. Looking for correlations with CT and age, we found a significant negative correlation between them; as age increases, CT decreases in all of the ETDRS areas except in II, where there was also a negative correlation close to being significant (−0.264; *p* = 0.056). Other authors, such as Abbey et al. [34], have studied the correlation between CT and age in a healthy group, finding a strong negative correlation between CT and age (*p* <0.001) in a group of patients between 22 and 89 years old.

A negative correlation was also found between RT and CT in the TE area (−0.305; *p* = 0.028), the C area (−0.287; *p* = 0.039), and the NI area (−0.405; *p* = 0.003), suggesting that if the retina increases its thickness, CT diminishes. There are studies, both clinical and experimental, as well as histopathological, which suggest a relationship between choroidal involvement and DR progression. Choroidal abnormalities have been described in DM, including the presence of microaneurysms, dilatations, closures, or capillary modification at the choriocapillaris level with increased vascular tortuosity, capillary loss, areas of nonperfusion, and choroidal neovascularization [17,35,36,37]. In diabetic patients with different degrees of DR, studies have shown a thinning in CT in subjects with proliferative DR or DME and fewer changes in nonproliferative DR stages or in the absence of DR signs. Alteration in the retinal vascular integrity, breakage of the blood-retinal barrier, or hemodynamic anomalies cause changes in CT in murine DM rodents [38,39]. The relationship between DR and diabetic choroidopathy is not clearly defined [40]. However, it is well known that the choroid supplies oxygen and nutrients to the outer retina, and any change or damage in its structure or a diminution in its vascularization could affect RT, causing hypoxia with the onset of retinal lesions or progression of existing DR to DME. However, it is not well known whether CT decrease occurs prior to the appearance of DR signs or whether DR lesions are associated with reduced CT.

Future prospective and longitudinal studies will be required to confirm these findings with a larger sample of diabetic patients, with different DR stages and with both DM1 and DM2, since they can behave in a different manner related to age, time of DM evolution, and other factors. Therefore, understanding the retina–choroid interactions as diabetes progresses and the physiopathological mechanisms involved in DR progression could help to optimize the management of this disease with adapted interventions depending on the degree or DR course.

## 5. Conclusions

In conclusion, DM2 patients with moderate DR have no changes with regard to CT. CT and RT decrease with age in DM2 patients and show a negative correlation between them in the central and horizontal parafoveal areas of the ETDRS grid, thereby indicating the thinning of CT and the thickening of RT.

## Figures and Tables

**Table 1:** Metabolic characteristics of type 2 diabetic (DM2) patients related to the duration and metabolic control of the disease. Abbreviations: HbA1c, glycosylated hemoglobin; HDL, high-density lipoprotein; LDL, low-density lipoprotein; TG, triglyceride levels; GF, glomerular filtration; SD, standard deviation. HbA1c values are expressed in a percent, cholesterol, TG, and creatine values in mg/dL and GF in mL/min.

DM2 Group	Mean	SD
Disease evolution time (years)	2.50	2.88
HbA1c (%)	7.58	1.29
Cholesterol (mg/dL)	148.04	33.18
HDL (mg/dL)	47.83	15.21
LDL (mg/dL)	71.47	23.09
TG (mg/dL)	122.24	51.71
GF (mL/min)	73.57	20.52
Creatine (mg/dL)	1.05	0.49

**Table 2:** Mean, standard deviation (SD), and statistical significance (*p*-value) of best corrected visual acuity (BCVA) in the LogMAR scale, spherical equivalent (SE) in diopters (D), axial length (AL) in mm, and intraocular pressure (IOP) in mmHg between the control and type 2 diabetes mellitus (DM2) groups. Differences that reached statistical significance (*p* < 0.05) are shown in bold.

	Control Group		DM2 Group		
	Mean	SD	Mean	SD	*p*
BCVA (LogMAR)	0.04	0.05	0.12	0.17	**<0.001**
SE (D)	0.03	1.58	0.37	1.70	0.110
AL (mm)	23.73	1.46	23.23	0.84	0.080
IOP (mmHg)	15.30	2.89	14.76	2.49	0.676

## Data Availability

The data presented in this study are available within the article.

## References

[1] Williams R., Karuranga S., Malanda B., Saeedi P., Basit A., Besançon S., Bommer C., Esteghamati A., Ogurtsova K., Zhang P. (2020). Global and regional estimates and projections of diabetes-related health expenditure: Results from the International Diabetes Federation Diabetes Atlas, 9th edition. Diabetes Res. Clin. Pract..

[2] Lupidi M., Coscas G., Coscas F., Fiore T., Spaccini E., Fruttini D., Cagini C. (2017). Retinal Microvasculature in Nonproliferative Diabetic Retinopathy: Automated Quantitative Optical Coherence Tomography Angiography Assessment. Ophthalmic Res..

[3] Couturier A., Mané V., Bonnin S., Erginay A., Massin P., Gaudric A., Tadayoni R. (2015). Capillary plexus anomalies in diabetic retinopathy on optical coherence tomography angiography. Retina.

[4] Orduna-Hospital E., Perdices L., Sanchez-Cano A., Acha J., Cuenca N., Pinilla I. (2020). Choroidal changes of long-term type 1 diabetic patients without retinopathy. Diagnostics.

[5] Abadia B., Suñen I., Calvo P., Bartol F., Verdes G., Ferreras A. (2018). Choroidal thickness measured using swept-source optical coherence tomography is reduced in patients with type 2 diabetes. PLoS ONE.

[6] Nickla D.L., Wallman J. (2010). The multifunctional choroid. Prog. Retin. Eye Res..

[7] Slakter J.S., Yannuzzi L.A., Guyer D.R., Sorenson J.A., Orlock D.A. (1995). Indocyanine-green angiography. Curr. Opin. Ophthalmol..

[8] Invernizzi A., Pellegrini M., Cornish E., Yi Chong Teo K., Cereda M., Chabblani J. (2020). Imaging the Choroid: From Indocyanine Green Angiography to Optical Coherence Tomography Angiography. Asia Pacific J. Ophthalmol. Philadelphia Pa..

[9] Meleppat R.K., Ronning K.E., Karlen S.J., Burns M.E., Pugh E.N., Zawadzki R.J. (2021). In vivo multimodal retinal imaging of disease-related pigmentary changes in retinal pigment epithelium. Sci. Rep..

[10] Pinilla I., Sanchez-Cano A., Insa G., Bartolomé I., Perdices L., Orduna-Hospital E. (2020). Choroidal differences between spectral and swept-source domain technologies. Curr. Eye Res..

[11] Orduna-Hospital E., Sanchez-Cano A., Perdices L., Acha J., Lopez-Alaminos E.M., Pinilla I. (2021). Changes in retinal layers in type 1 diabetes mellitus without retinopathy measured by spectral domain and swept source OCTs. Sci. Rep..

[12] Boned-Murillo A., Diaz-Barreda M.D., Ferreras A., Bartolomé-Sesé I., Orduna-Hospital E., Montes-Rodríguez P., Ascaso J., Pinilla I. (2021). Structural and functional findings in patients with moderate diabetic retinopathy. Graefe’s Arch. Clin. Exp. Ophthalmol..

[13] Maruko I., Iida T., Sugano Y., Oyamada H., Akiba M., Sekiryu T. (2012). Morphologic analysis in pathologic myopia using high-penetration optical coherence tomography. Investig. Ophthalmol. Vis. Sci..

[14] Maruko I., Iida T., Sugano Y., Ojima A., Sekiryu T. (2011). Subfoveal choroidal thickness in fellow eyes of patients with central serous chorioretinopathy. Retina.

[15] Maruko I., Iida T., Sugano Y., Oyamada H., Sekiryu T., Fujiwara T., Spaide R.F. (2011). Subfoveal choroidal thickness after treatment of vogt–koyanagi–harada disease. Retina.

[16] (1991). Fundus photographic risk factors for progression of diabetic retinopathy. ETDRS report number 12. Early Treatment Diabetic Retinopathy Study Research Group. Ophthalmology.

[17] Kim J.T., Lee D.H., Joe S.G., Kim J.G., Yoon Y.H. (2013). Changes in choroidal thickness in relation to the severity of retinopathy and macular edema in type 2 diabetic patients. Investig. Ophthalmol. Vis. Sci..

[18] Wang H., Tao Y. (2019). Choroidal structural changes correlate with severity of diabetic retinopathy in diabetes mellitus. BMC Ophthalmol..

[19] Haddad N.M.N., Sun J.K., Abujaber S., Schlossman D.K., Silva P.S. (2014). Cataract surgery and its complications in diabetic patients. Semin. Ophthalmol..

[20] Sandra Johanna G.P., Antonio L.A., Andrés G.S. (2019). Correlation between type 2 diabetes, dry eye and Meibomian glands dysfunction. J. Optom..

[21] Bikbova G., Oshitari T., Tawada A., Yamamoto S. (2012). Corneal changes in diabetes mellitus. Curr. Diabetes Rev..

[22] Bron A.J., de Paiva C.S., Chauhan S.K., Bonini S., Gabison E.E., Jain S., Knop E., Markoulli M., Ogawa Y., Perez V. (2017). TFOS DEWS II pathophysiology report. Ocul. Surf..

[23] Ewing F.M., Deary I.J., Strachan M.W., Frier B.M. (1998). Seeing beyond retinopathy in diabetes: Electrophysiological and psychophysical abnormalities and alterations in vision. Endocr Rev..

[24] Sokol S., Moskowitz A., Skarf B., Evans R., Molitch M., Senior B. (1985). Contrast Sensitivity in Diabetics With and Without Background Retinopathy. Arch. Ophthalmol..

[25] Ismail G.M., Whitaker D. (1998). Early detection of changes in visual function in diabetes mellitus. Ophthalmic Physiol. Opt..

[26] Tiepei Z.H.U., Jin M.A., Yonghao L.I., Zheng Z. (2015). Association between retinal neuronal degeneration and visual function impairment in type 2 diabetic patients without diabetic retinopathy. Sci. China Life Sci..

[27] Lee M.W., Lee W.H., Ryu C.K., Kim T.Y., Lim H.B., Lee Y.H., Kim J.Y. (2020). Effects of prolonged type 2 diabetes on the inner retinal layer and macular microvasculature: An optical coherence tomography angiography study. J. Clin. Med..

[28] van Dijk H.W., Kok P.H., Garvin M., Sonka M., Devries J.H., Michels R.P., van Velthoven M.E., Schlingemann R.O., Verbraak F.D., Abramoff M.D. (2009). Selective loss of inner retinal layer thickness in type 1 diabetic patients with minimal diabetic retinopathy. Investig. Ophthalmol. Vis. Sci..

[29] Jiang J., Liu Y., Chen Y., Ma B., Qian Y., Zhang Z., Zhu D., Wang Z., Xu X. (2018). Analysis of changes in retinal thickness in type 2 diabetes without diabetic retinopathy. J. Diabetes Res..

[30] Lattanzio R., Brancato R., Pierro L., Bandello F., Iaccher B., Fiore T., Maestranzi G. (2002). Macular thickness measured by optical coherence tomography (OCT) in diabetic patients. Eur. J. Ophthalmol..

[31] Wang W., Liu S., Qiu Z., He M., Wang L., Li Y., Huang W. (2020). Choroidal thickness in diabetes and diabetic retinopathy: A swept source OCT study. Investig. Ophthalmol. Vis. Sci..

[32] Laíns I., Talcott K.E., Santos A.R., Marques J.H., Gil P., Gil J., Figueira J., Husain D., Kim I.K., Miller J.W. (2018). Choroidal thickness in diabetic retinopathy assessed with swept-source optical coherence tomography. Retina.

[33] Horváth H., Ecsedy M., Kovács I., Sándor G.L., Mallár K., Czakó C., Nagy Z.Z., Somogyi A. (2020). [Choroidal thickness changes in patients with diabetes]. Orv. Hetil..

[34] Abbey A.M., Kuriyan A.E., Modi Y.S., Thorell M.R., Nunes R.P., Goldhardt R., Yehoshua Z., Gregori G., Feuer W., Rosenfeld P.J. (2015). Optical coherence tomography measurements of choroidal thickness in healthy eyes: Correlation with age and axial length. Ophthalmic Surg. Lasers Imaging Retina.

[35] Melancia D., Vicente A., Cunha J.P., Abegão Pinto L., Ferreira J. (2016). Diabetic choroidopathy: A review of the current literature. Graefes Arch. Clin. Exp. Ophthalmol..

[36] Esmaeelpour M., Považay B., Hermann B., Hofer B., Kajic V., Hale S.L., North R.V., Drexler W., Sheen N.J. (2011). Mapping choroidal and retinal thickness variation in type 2 diabetes using three-dimensional 1060-nm optical coherence tomography. Investig. Ophthalmol. Vis. Sci..

[37] Vujosevic S., Martini F., Cavarzeran F., Pilotto E., Midena E. (2012). Macular and peripapillary choroidal thickness in diabetic patients. Retina.

[38] Cunha-Vaz J., Faria de Abreu J.R., Campos A.J. (1975). Early breakdown of the blood-retinal barrier in diabetes. Br. J. Ophthalmol..

[39] Ciulla T.A., Harris A., Latkany P., Piper H.C., Arend O., Garzozi H., Martin B. (2002). Ocular perfusion abnormalities in diabetes. Acta Ophthalmol. Scand..

[40] Rayess N., Rahimy E., Ying G.S., Bagheri N., Ho A.C., Regillo C.D., Vander J.F., Hsu J. (2015). Baseline choroidal thickness as a predictor for response to anti-vascular endothelial growth factor therapy in diabetic macular edema. Am. J. Ophthalmol..

